# A syringeable immunotherapeutic hydrogel enhances T cell immunity via *in-situ* activation of STING pathway for advanced breast cancer postoperative therapy

**DOI:** 10.3389/fimmu.2025.1523436

**Published:** 2025-03-19

**Authors:** Baozhen Zhang, Min Li, Jiahua Ji, Xinghui Si, Xiaojiao Yin, Guofeng Ji, Liqun Ren, Haochen Yao

**Affiliations:** ^1^ Department of Experimental Pharmacology and Toxicology, School of Pharmaceutical Sciences, Jilin University, Changchun, China; ^2^ Key Laboratory of Polymer Ecomaterials, Changchun Institute of Applied Chemistry, Chinese Academy of Sciences, Changchun, China; ^3^ Department of Gynecologic Oncology, Gynecology and Obstetrics Center, the First Hospital of Jilin University, Changchun, China; ^4^ Department of General Surgery, Xuanwu Hospital, Capital Medical University, Beijing, China; ^5^ Hepatobiliary and Pancreatic Surgery Department, General Surgery Center, First Hospital of Jilin University, Changchun, China

**Keywords:** cancer immunotherapy, hydrogel, STING pathway, breast cancer, drug delivery

## Abstract

Complete surgical resection of advanced breast cancer is highly challenging and often leaves behind microscopic tumor foci, leading to inevitable relapse. Postoperative formation of the immunosuppressive tumor microenvironment (TME) reduces the efficacy of immunotherapies against residual tumors. Although cytotoxic chemotherapeutics exert the capacity to intensify cancer immunotherapy via immunogenic cell death (ICD) effects, systemically administered chemo agents often cannot access residual tumor sites, and fail to elicit antitumor immune responses. Herein, we present a novel syringeable immunotherapeutic hydrogel (SiGel@SN38/aOX40) loaded with the DNA-targeting chemotherapeutic 7-ethyl-10-hydroxycamptothecin (SN38) and the anti-OX40 agonist antibody (aOX40). The sustained in-site release of SN38 and aOX40 activate the stimulator of interferon genes (STING) pathway, intensify type I interferons expression, synergistically facilitate dendritic cell (DC) activation, and initiate persistent T cell mediated immune responses within the surgical resection bed that eliminate residual tumors with no tumor recurrence in 120 days. Collectively, our designed SiGel@SN38/aOX40 induces robust and long-lasting tumoricidal immunity following breast cancer resection and exhibit immense potential for clinical translation.

## 1 Introduction

Breast cancer ranks the main cause of cancer-related mortality among women worldwide (1). Despite multiple advancements in treatment modalities, surgical resection maintains its strong position for treating breast cancer (2). However, the complete surgical resection of advanced breast cancer is exceedingly difficult, often leaving behind microscopic tumor foci and inevitably results in lethal relapse. Chemotherapy and radiotherapy are often applied to eliminate residual tumors after surgery and partly prevent the local recurrence, but these therapies often cause toxicities and severe side effects (3, 4). Multiple cancer immunotherapy strategies have revolutionized the treatment of solid tumors (5–7). However, breast cancer is a poorly immuno-genic and ‘cold’ tumor, which is characterized as relatively low mutational burden and inadequate infiltration of antitumor T cells, resulting in a low-response rate of postsurgical cancer immunotherapy (8–10). Recently, convincing evidence indicates that certain chemotherapeutic agents can enhance anticancer immunotherapy via immunogenic cell death (ICD) effects (11, 12). However, systemically administered chemotherapeutic agents often cannot access the sites of residual tumor, and fail to elicit antitumor immune responses. Additionally, conventional systemic chemotherapy can cause systemic and intratumoral lymphodepletion, resulting in immune suppression (13). Therefore, a novel immunotherapeutic strategy that can induce a potent post-surgical antitumor response with minimized toxicity is highly clinical required.

The postoperative formation of immunosuppressive tumor microenvironment (TME), which is characterized by the enrichment of immune suppressors and a lack of cytotoxic T lymphocyte infiltration, diminishes the antitumor efficacy of immunotherapies for residual tumor (14). Biomaterials-based local immunotherapy represents a promising strategy for preventing local tumor recurrence after surgery (15–18). Concentrating ICD-inducing chemotherapeutic agents could harnesses the abundant tumor-associated antigens derived from residual malignant cells at the tumor resection bed to elicit tumor-specific antitumor immunity while minimizing the systemic toxicity of chemotherapy (14). This suggested the possibility of biomaterials loaded with chemotherapeutic agents and immunostimulatory adjuvants for postoperative immunotherapy of breast cancer. In addition, identifying suitable drug combinations is critical for postoperative management of breast cancer. Activation of the stimulator of interferon genes (STING) pathway could induce type I interferons (IFNs) and other pro-inflammatory cytokines expression, further induce the maturation and activation of dendritic cells (DCs) for antigen presentation as well as increase the recruitment of cytotoxic T cells, thereby initiating a robust innate immune response (19–21). The DNA-targeting chemotherapeutic agent 7-ethyl-10-hydroxycamptothecin (SN38) was reported not only induce ICD but also activate the STING pathway and trigger type-I-IFN-driven antitumor immunity (22–24). The anti-OX40 antibody (aOX40) functions as an agonist that activates OX40, a receptor in the tumor necrosis factor receptor superfamily. Upon activation, OX40 engages intracellular pathways that promote T cell survival, especially during the activation of T-cell receptors, thereby enhancing antitumor immune responses (25–28).

**Scheme 1:**
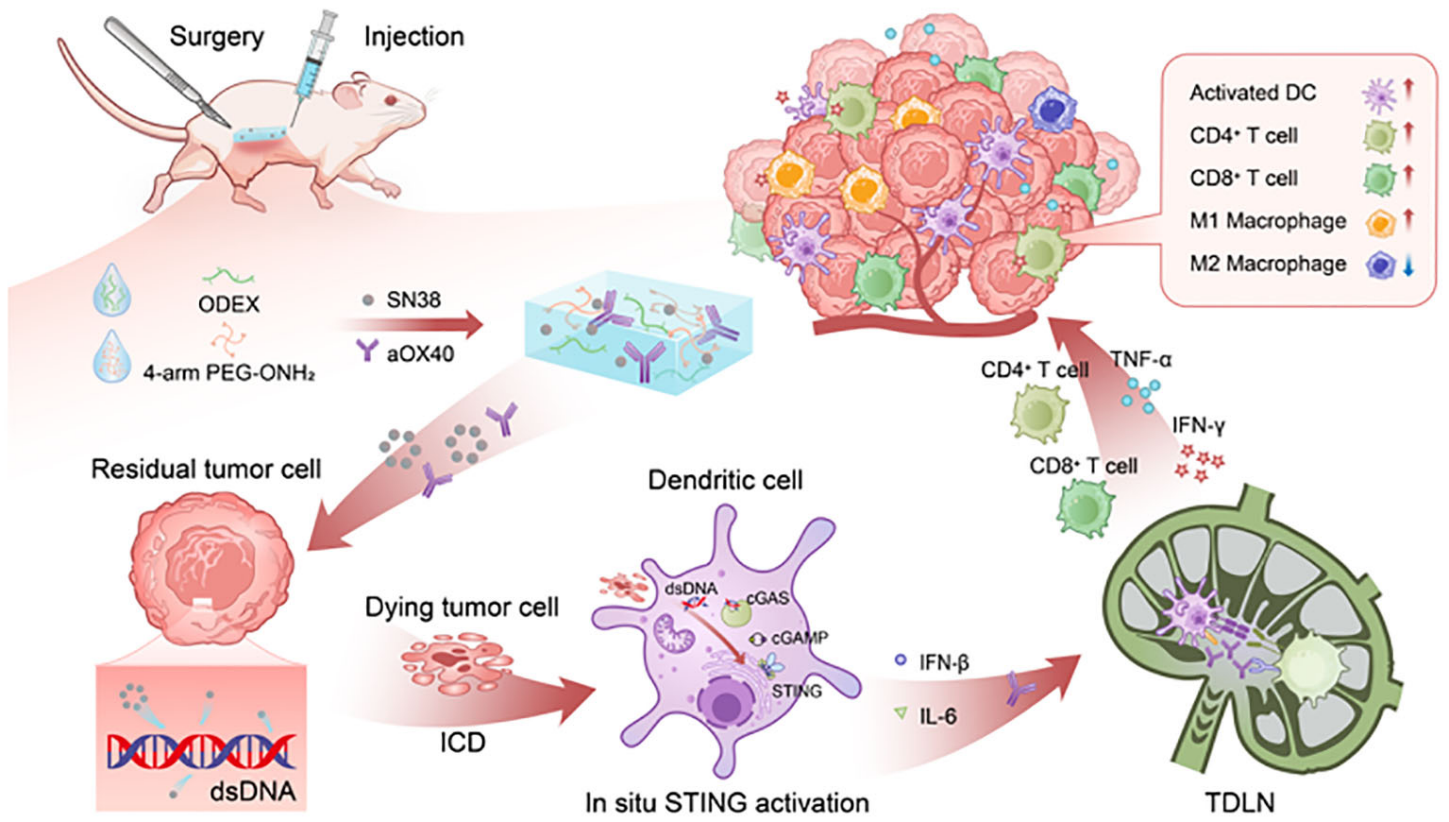
Schematic illustration of the prepared SiGel@SN38/aOX40 stimulates robust and durable tumoricidal immunity for advanced breast cancer postoperative therapy. The sustained, in-site release of SN38 and aOX40 activate STING pathway, intensify the expression of type I IFNs, multimodally facilitate DCs maturation and activation, and synergistically initiate persistent T cell mediated immune responses within the surgical resection bed, thereby eliminating residual tumors and preventing local recurrence with reduced systemic toxicity.

Herein, we designed a novel syringeable immunotherapeutic hydrogel (SiGel@SN38/aOX40) with *in situ* gelation and tissue adhesion capabilities for postoperative breast cancer immunotherapy based on our previously described technique (14, 15). The hydrogel was fabricated by cross-linking 4-arm polyethylene glycol hydroxylamine (4-arm PEG-ONH_2_) and oxidized dextran (ODEX) through oxmide bonds and co-loaded with SN38 and aOX40. The sustained in-site release of SN38 and aOX40 activate STING pathway, intensify the expression of type I IFNs, and multimodally facilitate DCs maturation and activation, and synergistically initiate persistent T cell mediated immune responses within the surgical resection bed that eliminate residual tumors and inhibits the local recurrence while minimizing systemic toxicity (**Scheme 1**). Our designed syringeable immunotherapeutic hydrogel modulates immunosuppressive TME induced by surgery and residual tumor, stimulates robust and long-lasting tumoricidal immunity after breast cancer surgical resection and holds significant potential for clinical application.

## 2 Materials and methods

### 2.1 Materials

SN38 was bought from Tokyo Chemical Industry (TCI) Co., Ltd (Tokyo, Japan). Anti-OX40 antibody was purchased from Bio X Cell (NH, USA). Rat IgG (Catalog: SP032) and Cell Counting Kit-8 (CCK-8) were bought from Solarbio Science & Technology Co., Ltd (Beijing, China). The 4-arm PEG-ONH_2_ (Mw = 10 kDa) was purchased from Jenkem Technology Co., Ltd (Beijing, China). ELISA kits (IFN-β, IL-6, INF-γ, TNF-α) were purchased from Elabscience Biotechnology Co., Ltd (Wuhan, China). Antibodies used for Western Blot were bought from Cell Signaling Technology Co., Ltd (MA, USA). BCA protein assay kit was obtained from Thermal Fisher Scientific Co., Ltd (MA, USA). GM-CSF and IL-4 were obtained from Beyotime Biotechnology Co., Ltd (Shanghai, China). All antibodies used for flow cytometry were obtained from BD Biosciences Co., Ltd (San Jose, CA, USA) and BioLegend Co., Ltd (San Diego, CA, USA).

### 2.2 Cell lines and animal models

The murine breast cancer cells (E0771 and 4T1) were obtained from BeNa Culture Collection (Beijing, China). Bone marrow derived dendritic cells (BMDCs) were harvested according to Son’s method (29). All the cells incubated at 37°C in culture medium at an atmosphere of 5% CO_2_.

Female C57BL/6 and BALB/c mice were bought from Beijing Vital River Laboratory Animal Technology Co., Ltd (Beijing, China). Female SD rats were obtained from Liaoning Changsheng biotechnology Co., Ltd (Liaoning, China). E0771 cells (1 × 10^6^ cells per mouse) were injected subcutaneously into the right flank of female C57BL/6 mice (6–8 weeks) to generate the subcutaneous E0771 tumor mouse model.

### 2.3 Preparation and characterization of syringeable immunotherapeutic hydrogel

The SiGel was prepared by cross-linking 4-arm PEG-ONH_2_ and ODEX through oxmide bonds at a weight ratio of 6% (w/w) based on our previously reported methods (14, 15). 400 μg SN38 was dissolved in DMSO (10 μL), and the solution was added to 4-arm PEG-ONH_2_ solution. Then the obtained hydrogel was named as SiGel@SN38. Similarly, aOX40 (15 μg) in solution was solubilized in 4-arm PEG-ONH_2_ solution to obtain SiGel@aOX40. The combinations of SN38 and aOX40 was designated as SiGel@SN38/aOX40.

The morphological characteristics of the lyophilized SiGel was assessed by scanning electron microscope (SEM, JSM-7000F, JEOL Ltd, Tokyo, Japan). Rheological analysis was performed according to our previously reported test methodology (15).

### 2.4 Assessment of the SiGel’s safety

The safety of the SiGel was evaluated by randomly assigning female C57BL/6 mice to two groups: the SiGel implant group and the PBS (surgery-only) control group. Peripheral blood samples were collected on days three and seven post-surgery. Serum IL-6 levels and complete blood counts were measured using Elisa assay kits, following the manufacturer’s instructions.

### 2.5 *In Vivo* and *In Vitro* degradation analysis

To evaluate *in vivo* degradation, female C57BL/6 mice were subcutaneously injected with SiGel into their flanks. Mice were euthanized at predetermined time points (7, 14, and 21 days), and the remaining SiGel samples were collected and documented through imaging.

For the *in vitro* degradation study, SiGel was immersed in PBS buffer (pH 7.4) and incubated at 37°C with continuous shaking at 90 rpm. The residual weight of SiGel was measured at predetermined time points.

### 2.6 *In Vitro* release of SN38 and aOX40 from SiGel

SiGel loaded with SN38 or IgG-Cy5 (a fluorescent analog representing the aOX40 antibody) was incubated in 2 mL of PBS (pH 7.4) at 37°C with constant shaking at 90 rpm. At predetermined intervals, the release medium was collected and replaced with fresh PBS. The concentration of SN38 was quantified using UV-Vis spectroscopy at 378 nm, while fluorescence spectroscopy ex = 649 nm, em = 670 nm) was used to measure the release of IgG-Cy5.

### 2.7 *In Vitro* cytotoxicity assay

The cytotoxic effects of free SN38 and SiGel@SN38 on E0771 cells, 4T1 cells, and BMDCs were assessed *in vitro* using CCK-8 assay kits. Briefly, tumor cells (4000 cells per well) and BMDCs (15000 cells per well) were seeded in 96-well plates and incubated overnight. Then the medium was removed and replaced with new media containing medicines. Following a 12-hour or 48-hour incubation, 20 μL of CCK-8 solution was added to each well. After a 2-hour incubation, absorbance was measured using a TECAN microplate reader. Cell viability (%) was calculated as the percentage of treated cells relative to the untreated control group.

### 2.8 *In Vivo* antitumor efficiency in E0771 incomplete resection tumor model

Female C57BL/6 mice bearing E0771 tumors (approximately 200–300 mm³) were prepared as described. In order to simulate the clinical state of incomplete tumor resection, approximately 90% of the tumor volume was surgically removed. The mice were then randomly assigned to 6 groups: untreated control (G1), SiGel (hydrogel without drug, G2), SiGel@SN38 (G3), SiGel@aOX40 (G4), Soluble@SN38/aOX40 (G5), and SiGel@SN38/aOX40 (G6). Each treatment was administered via injection into the tumor resection cavity. Body weight and residual tumor volume were continuously monitored.

### 2.9 Cytokine analysis

E0771 or 4T1 tumor cells were cultured in RPMI-1640 (containing 5 μM SN38) for 48 hours. The supernatant from these drug-pretreated tumor cells, designated as conditioned media (CM), was collected after centrifuging at 12,000 rpm for 5 minutes. BMDCs were then incubated for 12 hours in a mixture of 50% CM and 50% fresh RPMI-1640 media. Following incubation, the BMDC supernatant was gathered, centrifuged, and analyzed for cytokine content (IFN-β and IL-6) using ELISA kits.

The mice in each of the six groups had peripheral blood drawn at the end of the treatment. TNF-α and IFN-γ cytokine levels in serum and tumor were measured using ELISA kits.

### 2.10 Flow cytometry analysis

Tumors from the mice in each of the six groups were gathered at the ending of the trial. The tumors were cut into small pieces and lysed with tumor dissociation buffer (containing collagenase type IV, hyaluronidase, and DNase I). Then the supernatant was collected, filtered through a 300-mesh nylon filter, centrifuged and resuspended. Finally, fluorophore-conjugated antibodies were used to stain the resulting cell suspensions for 40 minutes on ice. A BD FACS Celesta flow cytometer was used to test the samples, and FlowJo software was used to analyze the results.

### 2.11 Western blot analysis

The supernatant from E0771 cells was collected as previously described and applied to treat BMDCs for 6 hours. Following this treatment, BMDCs were harvested and rinsed with 0.9% NaCl. RIPA lysis buffer was then added, and the samples were incubated on ice for 30 minutes. Proteins were extracted by centrifugation at 12,000 rpm for 5 minutes, and protein concentration was determined using a BCA protein assay kit. The extracted proteins were combined with loading buffer and heated to 100°C for 10 minutes. Protein separation was performed by SDS-PAGE using a Bio-Rad Mini gel electrophoresis system (Bio-Rad, USA), and the proteins were subsequently transferred onto PVDF membranes. Membranes were incubated overnight with specific primary antibodies on a shaker at 4°C. After washing the PVDF membranes five times, they were incubated with HRP-conjugated secondary antibodies for 1 hour. Finally, Western blot images were captured.

### 2.12 Statistical analysis

The mean ± standard deviation (S.D.) was used to display all data. Two treated groups were compared using two-tailed unpaired Student’s t-tests. *P* < 0.05 was used to test for significance.

## 3 Results

### 3.1 Preparation and characterization of SiGel

**Figure 1:**
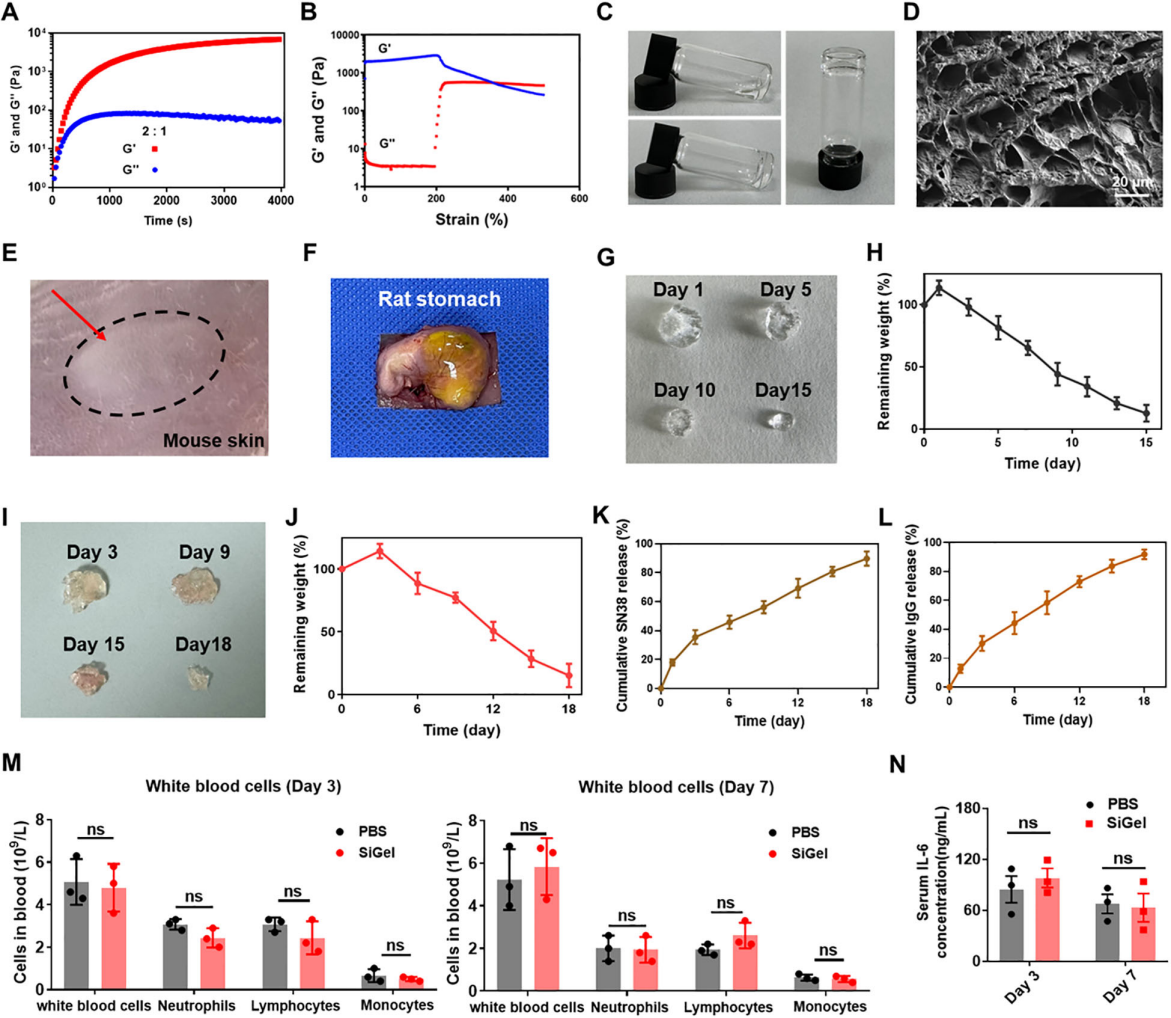
Preparation and characterization of SiGel. **(A, B)** Rheology properties of SiGel. **(C)** The photos of SiGel via a tube inverted test. **(D)** SEM images of lyophilized SiGel. Scale bar: 10 μm. **(E)** Subcutaneous injection of SiGel on BALB/C mouse after 5 min. **(F)** The images of SiGel injected on rat stomach though the syringe with 26 G needle. **(G, H)** *In vitro* degradation of SiGel under physiological conditions (*n* = 3). **(I, J)** *In vivo* degradation test of the SiGel (*n* = 3). **(K, L)** *In vitro* release profiles of SN38 and IgG from the SiGel *in vitro* (*n* = 3). **(M)** The complete blood count after on day 3 and day 7 (*n* = 3). **(N)** The IL-6 concentration in the serum on day 3 and day 7 (*n* = 3). Data are presented as means ± S.D. (^ns^ *P* > 0.05).

Based on our previous research, we synthesized SiGel by cross-linking 4-arm polyethylene glycol hydroxylamine (4-arm PEG-ONH_2_) and oxidized dextran (ODEX) at a 2:1 ratio, maintaining a solid content of 6% to ensure optimal injectability, synchronized degradation, and controlled release of the loaded agents (15, 30). Under these conditions, SiGel exhibited a storage modulus (G’) exceeding 6000 Pa and a loss modulus (G’’) of 35.8 Pa. Additionally, G’ decreased below G′′ at a strain of 370%, indicating the disruption of the hydrogel network at high strain (**Figures 1A, B**). The gelation properties of SiGel were confirmed via the tube inversion test (**Figure 1C**). Additionally, the SiGel also exhibited good shear-thinning characteristics *in vitro* and could be constantly injected without clogging by using a 26G needle (**Supplementary Figure 1**). SEM images exhibited that the lyophilized SiGel had a porous and interconnected structure with a diameter of ≈10–30 µm (**Figure 1D**).

We next investigated the injectability of the SiGel *in vivo*. SiGel was subcutaneously injected into female BALB/c mice. As shown in **Figure 1E**, the injected hydrogel quickly formed a bolus upon injection. Furthermore, the SiGel could firmly adhered to the surface of the rat stomach after injection, ensuring the SiGel firmly immobilized at the surgical site (**Figure 1F**).

### 3.2 Degradation and release characterization of SiGel

As depicted in **Figures 1G**, **H**, the SiGel experience a swelling at first and degraded gradually for over 15 days. For *in vivo* degradation assessment, SiGel was subcutaneously injected into the flanks of female C57BL/6 mice. At predetermined intervals (3, 9, 15, and 18 days), the mice were sacrificed, and the residual SiGel samples were collected and photographed. As shown in **Figure 1I**, **J**, *in vivo* degradation continued for > 18 days, indicating the great biodegradability of the SiGel. The sustained-release properties of the prepared SiGel ensure the sustained release of loaded drugs.

Due to its gradual degradation and diffusion mechanism, 89.8% of encapsulated SN38 and 92% of IgG-Cy5 were released in a sustained manner over 18 days (**Figures 1K, L**). The extended and sustained release of SN38 and aOX40 within the surgical area is expected to maximize synergistic antitumor efficacy.

### 3.3 Safety assessment of SiGel

The safety of prepared SiGel is crucial for clinical application. As shown in **Figures 1M**, **N**, no significant alterations were observed in white blood cell counts or serum IL-6 levels, indicating a favorable biocompatibility profile. The biosafety of the SiGel was further confirmed through *in vitro* cytotoxicity assay. As shown in **Supplementary Figure 2**, no obvious cytotoxicity was observed in murine 3T3 fibroblasts and human coronary artery endothelial cells (HCAECs) after 48-hour incubation with a high SiGel concentration (40mg/mL), demonstrating excellent cytocompatibility. Additionally, the SiGel-treated mice exhibited no signs of weight loss compared to the control group (**Supplementary Figure 3**).

### 3.4 Activation of the STING pathway by SiGel

**Figure 2:**
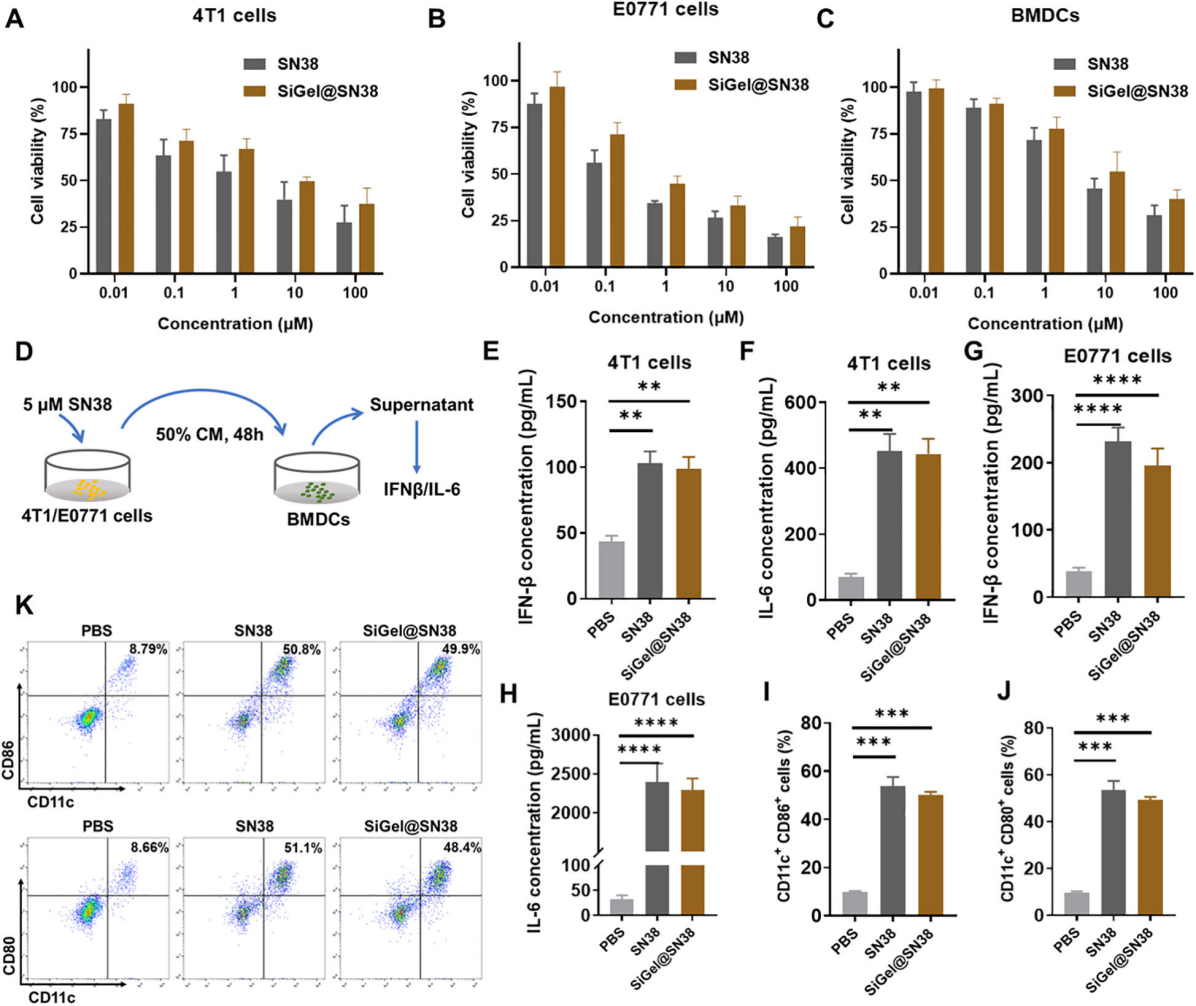
Evaluation of SiGel for activation of STING pathway. **(A–C)** *In vitro* cell cytotoxicity of free SN38 and SiGel@SN38 on 4T1 **(A)**, E0771 **(B)** at 48 h, and BMDCs **(C)** at 12 h (*n* = 4). **(D)** Schematic of cytokine secretion test. **(E–H)** IFN-β **(E, G)** and IL-6 **(F, H)** concentration in supernatants of cells (*n* = 3). **(I, J)** The expression of stimulatory molecule CD80 and CD86 on BMDCs surface after the incubation of PBS or free SN38 or SiGel@SN38 treated E0771 CM for 12 h (*n* = 3). **(K)** The representative flow cytometric quantification of CD86 and CD80 on BMDCs surface. Data are presented as means ± S.D. (**P < 0.01, ***P < 0.001, and ****P < 0.0001).

First, we determined the cytotoxicity of blank SiGel (hydrogel without drug), free SN38 and SiGel@SN38 (SiGel loaded with SN38) to E0771 cells, 4T1 cells and BMDCs using CCK-8 assay. As expected, blank SiGel exhibited no significant cytotoxicity toward E0771 cells, 4T1 cells and BMDCs, even at high concentration (40mg/mL) (**Supplementary Figure 4**). In contrast, both free SN38 and SiGel@SN38 displayed dose-dependent cytotoxicity against E0771 cells, 4T1 cells and BMDCs (**Figures 2A–C**). SN38 displayed stronger tumor cell inhibiting effects on E0771 cells than 4T1 cells. Intriguingly, SiGel@SN38 showed a weaker cytotoxic effect than free SN38, which may attribute to the gradual release of SN38 from hydrogel (**Figures 2A–C**).

Dendritic cells (DCs) exert a vital role in stimulating innate and adaptive immune responses (31). To assess the capacity of SiGel@SN38 to activate the STING pathway, we evaluated its effects on type I interferon (IFN) and pro-inflammatory cytokine secretion. STING activation induces type I IFNs and other pro-inflammatory cytokines via phosphorylation of tank-binding kinase 1 (TBK1) and interferon regulatory factor 3 (IRF3), thereby promoting DC activation, antigen presentation, and cytotoxic T-cell recruitment, ultimately enhancing antitumor immunity (32, 33). ELISA assays were conducted to measure IFN-β and IL-6 secretion following SiGel@SN38 treatment (**Figure 2D**). As shown in **Figures 2E–H**, both free SN38 and SiGel@SN38 treatment significantly increased IFN-β and IL-6 secretion. Western blot analysis further confirmed the activation of STING-related signaling pathways, showing increased phosphorylation of STING, IRF3, and TBK1 within 4 hours of SN38 or SiGel@SN38-conditioned medium (CM) treatment (**Supplementary Figure 5**). These results presented here confirmed that SiGel@SN38 effectively activates the STING pathway. Next, we further evaluated the ability of SiGel@SN38 to promote DCs maturation and activation using flow cytometry. As shown in **Figures 2I–K**, BMDCs treated with CM from SiGel@SN38-treated E0771 cells exhibited a significant increase in the expression of the stimulatory molecules CD80 and CD86. Collectively, these findings demonstrate that the sustained release of SN38 from SiGel effectively stimulates the STING pathway and induces a robust antitumor immune response *in vitro*.

### 3.5 *In Vivo* antitumor efficiency in E0771 incomplete resection tumor model

**Figure 3:**
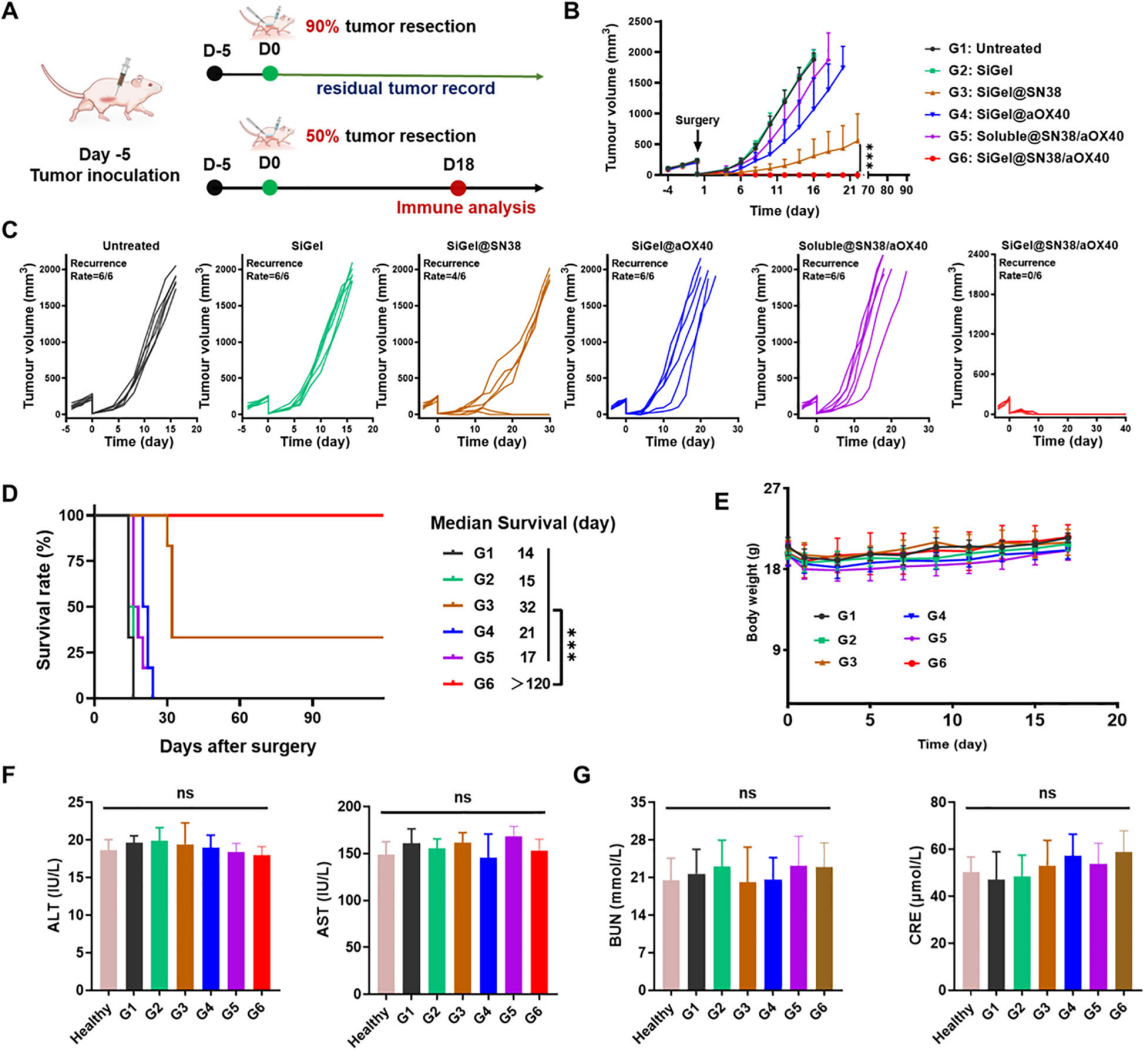
*In Vivo* antitumor efficiency in E0771 incomplete resection tumor model. **(A)** The schematic of the treatment process. **(B–E)** Average **(B)** and individual **(C)** tumor growth kinetics in different groups (*n* = 6). **(D)** Survival curves of mice after different treatments (*n* = 6). **(E)** Weight changes in different groups (*n* = 6). **(F, G)** Serum levels of AST, ALT, BUN and CRE in mice after different treatments and normal heathy mice (*n* = 3). Data are presented as means ± S.D. (^ns^ *P* > 0.05, ***P < 0.001).

In order to simulate the clinical state of incomplete tumor resection, we established an incomplete tumor resection model by excising ~90% tumor volume when E0771 tumor on the female C57BL/6 mice reached a volume of 200–300 mm^3^ (**Figure 3A**). The mice were then randomly divided into 6 groups: untreated (G1), SiGel (hydrogel without drug, G2), SiGel@SN38 (G3), SiGel@aOX40 (G4), Soluble@SN38/aOX40 (G5), and SiGel@SN38/aOX40 (G6), with corresponding drug injected into the tumour resection cavity, respectively. As shown in **Figures 3B–D**, rapid tumor relapse was observed in the untreated (control) group, with a median survival of only 14 days post-surgery. Consistent with the cytotoxicity results, SiGel alone did not show therapeutic effects and the median postoperative survival time was similar to that of untreated group. SiGel@aOX40 treatment exhibited minimal therapeutic effect and fail to supress tumor recurrence and improve postoperative outcome. SiGel@SN38 treatment showed moderate therapeutic effect and partially suppressed tumor growth, with a prolonged postoperative length of 32 days. Notably, SiGel@SN38/aOX40 dramatically suppressed the tumor growth and resulted in complete tumor eradication, with no tumor recurrence over the 120 days surveillance period. Intriguingly, the therapeutic effects of Soluble@SN38/aOX40 were not long-lasting and simply delay the time of the tumor relapse. Ultimately, all mice in the Soluble@SN38/aOX40 group relapsed, demonstrating the importance of encapsulating pharmaceuticals into hydrogels. These results indicated that our designed SiGel and loaded drug combination is necessary for trigging robust and durable tumoricidal immunity following breast cancer surgical resection.

Owing to the trauma caused by surgery and anesthesia, almost all treated mice experienced a body weight loss (≈ 15%) after surgery (**Figure 3E**). Then the treated mice across the groups soon returned to their initial body weight, indicating the safety of these treatments. In addition, AST, ALT, and BUN levels were measured to further assess the system toxicity of various treatments. As shown in **Figures 3F**, **G**, no significant liver or kidney dysfunction was observed in treated mice compared to healthy controls. These results demonstrated that our hydrogel-based treatment exhibits a favorable safety profile with minimal and controllable side effects.

**Figure 4:**
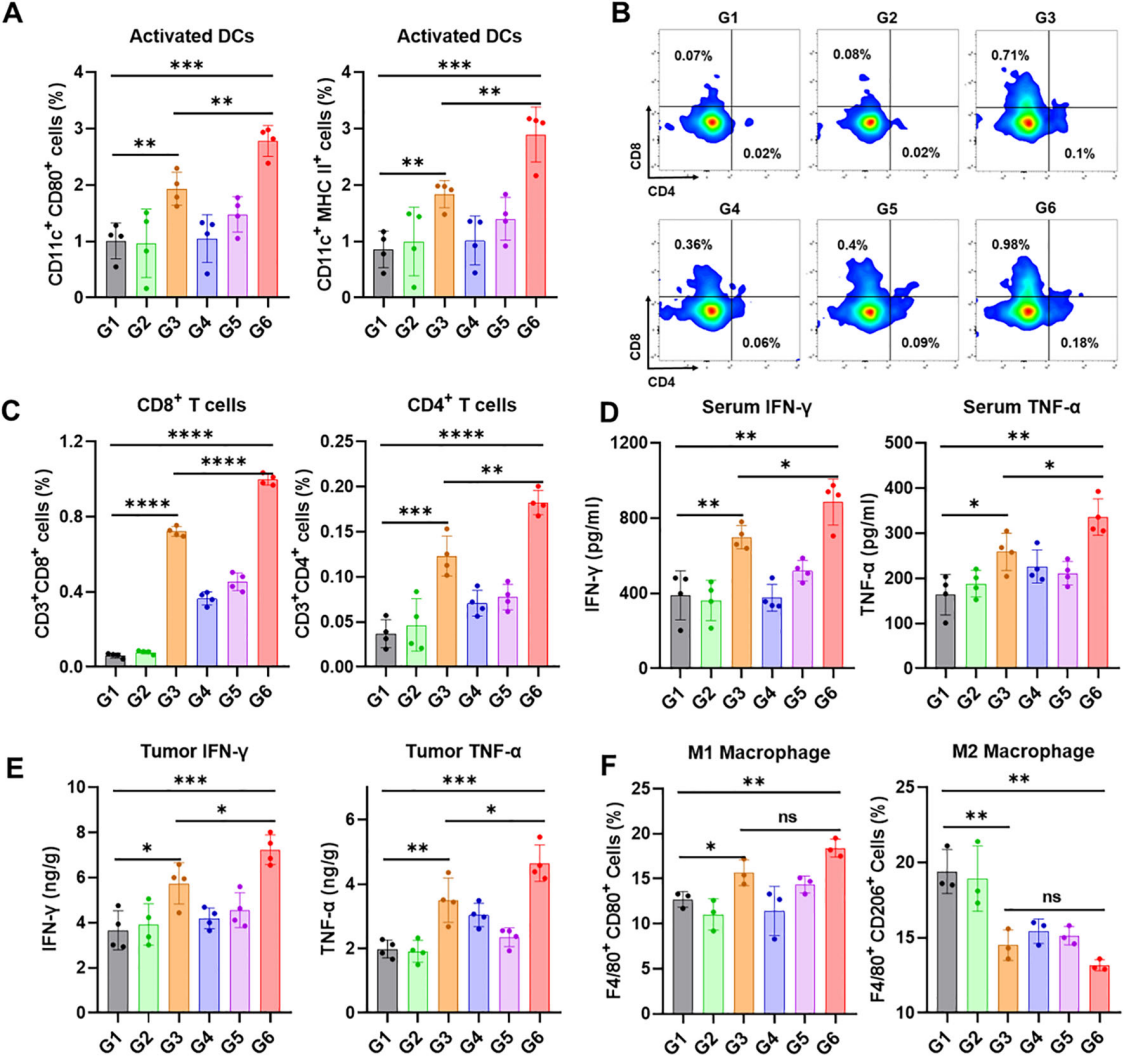
SiGel@SN38/aOX40 for triggering antitumour immune response. **(A)** Flow cytometry analysis of activated DCs in tumor tissues following various treatments (*n* = 4). **(B)** Representative flow cytometric analysis images of CD4**^+^** T cells and CD8**^+^** T cells. **(C)** Flow cytometry analysis of CD4**^+^** T cells and CD8**^+^** T cells cells in tumor tissues following various treatments (*n* = 4). **(D, E)** The level of IFN-γ and TNF-α cytokines in serum **(D)** and tumor **(E)** after various treatments (*n* = 4). **(F)** Flow cytometry analysis of macrophage in tumor tissues following various treatments (*n* = 3). G1: Untreated, G2: SiGel, G3: SiGel@SN38, G4: SiGel@aOX40, G5: Soluble@SN38/aOX40, G6: SiGel@SN38/aOX40. Data are presented as means ± S.D. (^ns^ *P* > 0.05, *P < 0.05, **P < 0.01, ***P < 0.001, and ****P < 0.0001).

To explore the potential mechanisms of the therapeutic effects after various treatments, another subcutaneous E0771 breast cancer model was used to examine the tumor immune microenvironment. In this model, only half of the tumor volume was resected to preserve sufficient tissue for analysis of infiltrating immune cells via flow cytometry at the end of the treatment. Activation of STING pathway could induce DCs maturation and activation, thereby promoting the recruitment of cytotoxic T cells from tumor draining lymph nodes. As expected, the proportion of mature DCs in the SiGel@SN38 and SiGel@SN38/aOX40 groups significantly increased (**Figure 4A**). Of note, the proportion of mature DCs was lower in the Soluble@SN38/aOX40 group, highlighting the necessity of sustained, *in situ* drug release from the hydrogel. Furthermore, SiGel@SN38/aOX40 treatment displayed the highest proportions of CD4**^+^** and CD8**^+^** T cells in comparison with other groups (**Figures 4B, C**). The secretion level of TNF-α and IFN-γ in serum and tumor were detected. As shown in **Figures 4D**, **E**, SiGel@SN38/aOX40 treatment significantly enhanced antitumor immunity by elevating pro-inflammatory cytokines. Extensive research has shown that tumor associated macrophages (TAM), the main tumor-infiltrating cells, play pivotal roles in tumor progression. Macrophages can differentiate into M1 phenotype or M2 phenotype. M2 phenotype exhibit pro-tumorigenic activities, whereas M1 phenotype play critical roles in antigen presentation and exert anti-tumorigenic function (34, 35). Reducing M2 macrophages or increasing M1 macrophages is crucial for enhancing T cell-mediated immunity (36). As shown in **Figure 4F**, SiGel@SN38/aOX40 treatment significantly increased M1 macrophages and reduced M2 macrophages within the tumor, indicating a reprogramming of the tumor immune microenvironment toward immune activation. These results evidenced that SiGel@SN38/aOX40 treatment effectively stimulates strong antitumor immunity *in vivo*.

## 4 Discussion

Despite multiple advancements in treatment modalities, surgical resection maintains its strong position for treating breast cancer. However, complete resection of advanced breast cancer is challenging and often leaves behind microscopic tumor foci, leading to local recurrence and distant metastasis. The therapeutic effects of chemotherapy and radiotherapy are limited, often resulting in toxicities and severe side effects (3, 4). Over the past decade, cancer immunotherapy has shown great promise in postoperative cancer treatment by activating systemic anticancer immunity (14, 15, 37, 38). However, breast cancer is considered a “cold tumor,” characterized by a low mutational burden and insufficient infiltration of antitumor T cells, resulting in a poor response to postsurgical immunotherapy (8–10). Therefore, a new immunotherapeutic strategy that can achieve robust post-surgical antitumor effects with minimized toxicity is highly clinical required.

Multiple publications have demonstrated that biomaterial-based local immunotherapy is a highly promising immunotherapeutic strategy for preventing local tumor recurrence after surgery (15–18). Herein, we designed a novel syringeable immunotherapeutic hydrogel (SiGel@SN38/aOX40) with *in situ* gelation and tissue adhesion capabilities for breast cancer postoperative immunotherapy based on our previously described technique (14, 15). The hydrogel was fabricated by cross-linking 4-arm PEG-ONH_2_ and ODEX through oxmide bonds and co-loaded with SN38 and aOX40. The SiGel exhibited excellent shear-thinning properties *in vitro*, enabling continuous injection without clogging using a 26G needle (**Supplementary Figure 1**). SEM images revealed that the lyophilized SiGel had a porous and interconnected structure with pore diameters of approximately 10–30 µm (**Figure 1D**). *In vivo* degradation of SiGel occurred over more than 18 days, with sustained release of the loaded drugs (**Figure 1D**). The prolonged and sustained release of SN38 and aOX40 antibody in the surgical area maximizes the synergistic antitumor efficacy. Additionally, the controlled and constant-rate release of the DNA-targeting chemotherapeutic agent SN38 from SiGel@SN38 effectively stimulate STING pathway, triggering robust antitumor immunity *in vitro*.

In this study, we established an incomplete breast cancer resection model to mimic the clinical condition of incomplete tumor resection. Tumor relapses appeared rapidly in the untreated group and SiGel (hydrogel without drug) groups, accompanied by a shorter postoperative survival period. In contrast, SiGel@SN38/aOX40 treatment dramatically suppressed E0771 tumor growth, leading to complete tumor eradication, with no recurrence over the 120-day observation period. Interestingly, the therapeutic effects of Soluble@SN38/aOX40 were transient, with all mice ultimately relapsing, highlighting the importance of the hydrogel-based delivery system for achieving durable tumoricidal immunity after breast cancer surgical resection. We further analyzed the immune microenvironment of residual tumor tissues 18 days post-treatment. As expected, the SiGel@SN38 and SiGel@SN38/aOX40 groups exhibited a significant increase in the proportion of activated DCs (**Figure 4C**). Additionally, SiGel@SN38/aOX40 treatment resulted in the highest proportions of CD4^+^ and CD8^+^ T cells, accompanied by elevated serum and tumor levels of IFN-γ and TNF-α compared to other groups. These findings provide strong evidence that our syringeable immunotherapeutic hydrogel activates the STING pathway, facilitates DC maturation and activation, and synergistically initiates persistent T cell-mediated immune responses within the surgical resection bed.

In summary, we designed a novel syringeable immunotherapeutic hydrogel constructed with dynamic reversible covalent bonds to achieve controlled, constant-rate in site release of DNA-targeting chemotherapeutic SN38 and aOX40. The sustained in-site release of SN38 and aOX40 activate STING pathway, synergistically facilitate dendritic cell (DC) activation, modulates immunosuppressive TME induced by surgery and residual tumor, and initiate persistent and durable tumoricidal immunity within the surgical resection bed. The designed SiGel@SN38/aOX40 shows immense potential for clinical application in advanced breast cancer postoperative therapy.

## Data Availability

The original contributions presented in the study are included in the article/**Supplementary Material**. Further inquiries can be directed to the corresponding author/s.
