# Supplementary material for: A syringeable immunotherapeutic hydrogel enhances T cell immunity via in-situ activation of STING pathway for advanced breast cancer postoperative therapy
Source: Front Immunol. 2025 Mar 19;16:1523436. doi: 10.3389/fimmu.2025.1523436 (PMC11961417; doi:10.3389/fimmu.2025.1523436)
Supplement: Supplementary file 1 [file DataSheet1.docx]

Supplementary Material

**A syringeable immunotherapeutic hydrogel enhances T cell immunity via in-situ activation of STING pathway for advanced breast cancer postoperative therapy**

Baozhen Zhang^1^, Min Li^1^, Jiahua Ji^1^, Xiaojiao Yin^2*^, Guofeng Ji^3*^, Liqun Ren^1*^, and Haochen Yao^4^

^1^Department of Experimental Pharmacology and Toxicology, School of Pharmaceutical Sciences, Jilin University, Changchun, China

^2^Department of Gynecologic Oncology, Gynecology and Obstetrics Center, the First Hospital of Jilin University, Changchun, China.

^3^Department of General Surgery, Xuanwu Hospital, Capital Medical University, Beijing, China

^4^Hepatobiliary and Pancreatic Surgery Department, General Surgery Center, First Hospital of Jilin University, Changchun, China

*** Correspondence:**

Xiaojiao Yin, 🖂 yinxj@jlu.edu.cn

Guofeng Ji, 🖂 jigf1227@163.com

Liqun Ren, 🖂 renlq@jlu.edu.cn

Keywords: cancer immunotherapy, hydrogel, STING pathway, breast cancer, drug delivery.


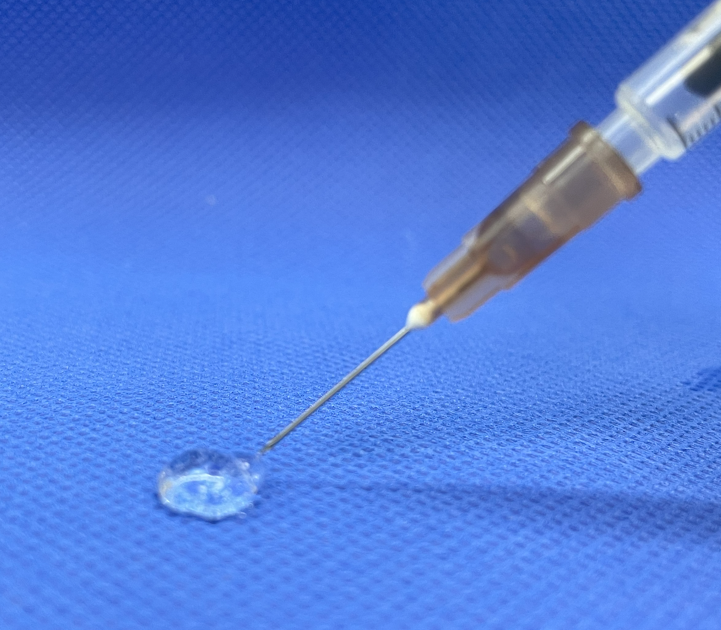


***Supplemental Figure 1*.** The SiGel could be continuously injected without clogging by using a 26G needle.

***Supplemental Figure 2*.** The cytotoxicity of SiGel on the 3T3 cells and HCAEC cells after incubation with various concentrations of SiGel for 48 h (*n* = 4).

***Supplemental Figure 3*.** The changes of body weight after injection the SiGel at day 0 on the female C57BL/6 mice or only surgery (PBS).


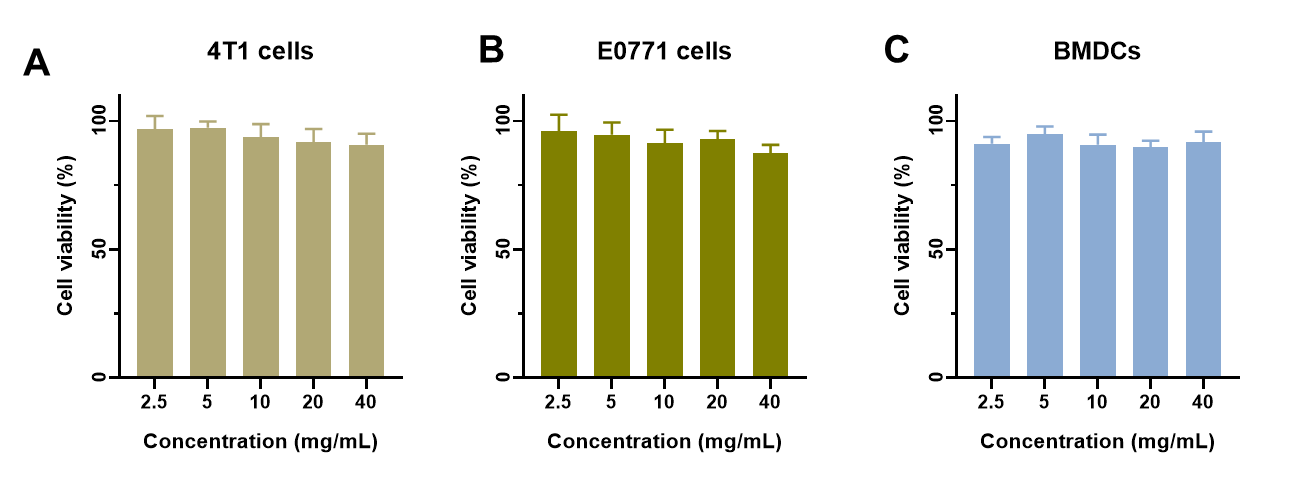


***Supplemental Figure 4*.** The cytotoxicity of SiGel on the 4T1 cells (A), E0771 cells (B), and BMDCs (C) after incubation with various concentrations of SiGel for 48 h (*n* = 5).


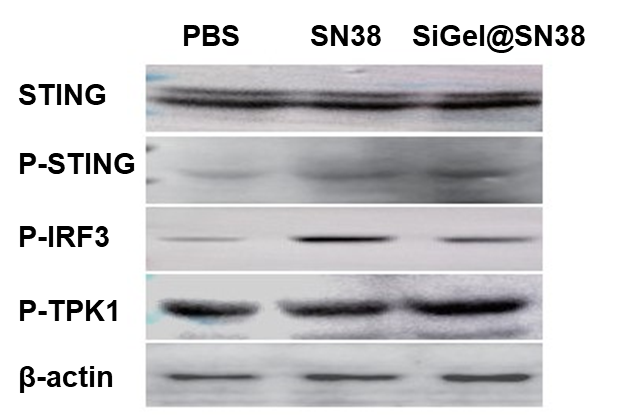


***Supplemental Figure 5*.**  Western blot images of proteins extracted from BMDCs after treated with PBS or E0771 free SN38 CM or E0771 SiGel@SN38 CM for 4 h.
